# Alcohol, Anti-HIV Drugs, and/or Hippuric Acid Deteriorate Cellular Stresses in Senescent Hepatocytes and Aging Murine Liver

**DOI:** 10.13188/2330-2178.1000059

**Published:** 2025-04-15

**Authors:** L Chen, M Kaypaghian, E Duran, C Ji

**Affiliations:** Department of Medicine, Keck School of Medicine of USC, University of Southern California, Los Angeles, California, USA

**Keywords:** Drug Use Disorders, Alcoholism, Cellular Stress, Senescence of Hepatocytes, Liver Aging and Injury

## Abstract

Liver disease has increased recently in aging people living with HIV and substance use disorders, and little is known about injurious effects of anti-HIV drugs, alcohol and other substances on cellular stress responses in senescent hepatocytes and aging liver. In this study, senescence of two liver cell lines: HepG2 and AML-12, was induced by hydrogen peroxide (H_2_O_2_) and confirmed by senescence makers including cyclin-dependent kinase inhibitor CKI (p21), senescence-associated β-galactosidase (SA-β-gal), or insulin-like growth factor 1 (IGF1). The cell and mouse models (2-month-old versus 23-month-old) were treated with ritonavir, lopinavir, ethanol, or cocaine derivative hippuric acid. Cell stress responses and stress-related metabolic proteins were evaluated. In non-senescent cells, treatments of alcohol, ritonavir and lopinavir alone or in combinations increased expression of endoplasmic reticulum stress related glucose-regulated protein 78 (GRP78) and C/EBP homologous protein (CHOP), had minor effects on glucose-regulated protein 75 (GRP75) and inositol 1,4,5-trisphosphate (IP3) receptor type 2 (IP3R2), ubiquitin specific protein 10 (USP10), USP17, USP20, lipogenic factor peroxisome proliferator-activated receptor gamma (PPARγ), Ras-related protein Rab-18 (RAB18), or Ras converting enzyme 1(RCE1). In the senescent cells, alcohol, ritonavir, lopinavir, and/or hippuric acid induced higher expression of GRP78, CHOP, IP3R2, USP17, USP20, PPARγ and RAB18, but reduced expression of GRP75, USP10, and RCE1. In the aged mice fed alcohol diet and the anti-HIV drugs, hepatic GRP78, CHOP, USP17, PPARγ, and triglycerides, number of senescent or dead hepatocytes, blood levels of alanine aminotransferase were significantly increased and RCE1 was reduced compared to young mice fed alcohol and the drugs. These results suggest that protein factors and responses that potentially function in ameliorating cellular stresses are undermined and protein factors that might cause cell dysfunctions or injury are exacerbated in the senescent hepatocytes and liver of aged mice treated with alcohol and anti-HIV drugs.

## Introduction

With successful antiretroviral treatment (ART), people living with HIV (PLWH) have extended their lifespan. More than half of PLWH in the United States are age 50 or older [1, 2]. Such extending lifespan into elderly years gives rise to age-related decline in major organs including the liver. The risk for liver pathology in PLWH without primary causes for the liver injury has been increased with aging [3, 4]. Moreover, elderly people tend to take more drugs and have substance use disorders such as alcoholism and cocaine use [5, 6]. Their liver may suffer more frequent or severe injury due to reduced liver capacity in detoxification of xenobiotics with age. For instance, activities of alcohol metabolism enzymes including alcohol dehydrogenase, acetaldehyde dehydrogenase and cytochrome P-4502E1 diminish with advancing age [7]. Alcohol consumption not only worsens alcohol-induced liver injury but also exacerbates drug toxicities in aging PLWH with ART due to rising drug-drug interactions and drug modifications by alcohol [8]. Besides alcohol, in PLWH with ART, certain anti-HIV drugs may also have impact on cellular senescence which may contribute to age-related decline in physiological functions. In organs other than liver, ART was reported to induce premature senescence and altered physiology in HUVECs [9, 10]. The anti-HIV protease inhibitors, ritonavir-boosted atazanavir, were demonstrated to induce cell growth arrest, multiple features of senescence, and senescence-associated secretory phenotypes [11]. However, whether ART contribute to senescence or aging of the liver is not clear and what effects of alcohol or substances on the liver of people with ART have not been investigated. Previously, we reported that in non-senescent liver cells or non-aged animal models, the anti-HIV drug ritonavir and lopinavir induced endoplasmic reticulum (ER) and Golgi dysfunctions, cellular stress responses and hepatic injury, and combination of the HIV drugs with alcohol exacerbated the organelle stress responses and injury [12–18]. In this study, we examined the effects of the anti-HIV drugs, combined with alcohol or a cocaine derivative, hippuric acid on cellular stress response and injury in senescent hepatocytes or aged liver of animal models, and document our findings.

## Materials and Methods

### Liver Cell Culture and Treatments

AML12 cells (#CRL-2254) and HepG2 cells (#HB-8065) were initially purchased from ATCC (USA). AML12 cells were maintained in DMEM/Hams F-12 (#10–092-CV, Corning) supplemented with 2% of fetal bovine serum (#A3160401, Gibco, ThermoFisher), insulin-transferrin-selenium (ITS) (#41400045, Gibco), 40 ng/ml dexamethasone (#D2915, Sigma-Aldrich), and 1% of penicillin-streptomycin (#15140–122, Gibco). HepG2 cells were maintained in Dulbecco’s Modified Eagle Medium (DMEM) (#11965–092, Gibco, ThermoFisher) supplemented with 10% of fetal bovine serum (FBS) (#A3160401, Gibco) and 1% of penicillin-streptomycin (#15140–122, Gibco) at 37 °C and 5% CO_2_ incubator. The medium was replaced every 3 days if not otherwise stated.

The senescence of the cells was established as described previously with minor modifications [15]. Briefly, AML12 cells were seeded at a density of 0.13 M/ml in 10 cm culture dish and allowed one night for attachment. The cells were treated with hydrogen peroxide(H_2_O_2_) (#H1009–5ML, Sigma-Aldrich) for a period of 6 days. In the first day, AML12 cells were incubated with 1mM H_2_O_2_ in DMEM/Hams F-12 (#10–092-CV, Corning) with 1% of penicillin-streptomycin (#15140–122, Gibco) for one hour and the medium was then refreshed without H_2_O_2_, which was followed by 750 μM H_2_O_2_ treatment in the same medium for one hour each day for the rest of 5 days. One day after the last H_2_O_2_ treatment, cells were replated to 6-well plates for drug treatments. The senescent model of HepG2 cells was established as described previously [16]. HepG2 cells were seeded at a density of 0.4 M/ml in 10 cm culture dish and allowed one night for attachment. Cells were treated with 300 μM H_2_O_2_ in complete medium for 3 consecutive days with medium refreshed every day. Cells were then recovered for 3 days before replating to 6-well plates for alcohol and/or drug treatments.

AML12 or HepG2 cells were incubated with complete medium with 25 μg/ml Ritonavir (#SML0491, Sigma-Aldrich), 25 μg/ml Lopinavir (#SML1222, Sigma-Aldrich), 2 μg/ml Tunicamycin (#T7765, Sigma-Aldrich), 0.1 uM Thapsigargin (#T9033, Sigma-Aldrich), 1 μg/ml Brefeld in A (#B5936–200Ul, Sigma-Aldrich), or 250 μM Hippuric acid (#112003, Sigma-Aldrich) alone or in combination for 24 hours.Dimethyl sulfoxide (DMSO, #D8418, Sigma-Aldrich; <0.5%) was used as vehicle control. For alcohol treatment of the cells, an acetaldehyde generating system (AGS) was applied [19, 20], which consisting of 0.08% of ethanol (#E7023; Sigma-Aldrich), 0.02 units of alcohol dehydrogenase (ADH, (#A3263; Sigma-Aldrich), and 2 mM of nicotinamide adenine dinucleotide (NAD+; #N6754, Sigma-Aldrich) continuously generates physiologically relevant acetaldehyde that mimics *in vivo* acetaldehyde production from the legal limit of blood alcohol concentration.

### Animal Models and Experiments

C57BL/6 mice were purchased from Jackson’s laboratory (stock# 000664 for 2-month-old and stock# 000665 for 23-month-old). The animals of each age were grouped into 4 groups and were fed *ad libitum* for 2 months with isocaloric control liquid diet (Dyets, Inc., Bethlehem, PA) (group 1), AIN-93G alcohol liquid diet containing 3.5% ethanol (Dyets, Inc., Bethlehem, PA) (group 2), the control diet with 25 μM of ritonavir and lopinavir (group 3), and the alcohol diet with 25 μM of ritonavir and lopinavir (group 4). The mice were euthanized for serum and liver tissues after the feeding. All animals were treated in accordance with the Guide for Care and Use of Laboratory Animals and the study was approved by the local animal care committee. Analyses for serum alanine aminotransferase (ALT), liver histology by hematoxylin and eosin staining (H&E), liver triglycerides, and immunohistochemistry & staining were described previously [12, 13, 17, 18]. Number of Ki-67 positive hepatocytes and hepatocytes with enlarged nuclei was counted in 3 microscope fields of each section of liver tissue with 200× original magnification. Isolation of primary mouse hepatocytes (PMH) was conducted by the Cell Culture Core of USC Research Center for Liver Disease. The livers of anesthetized mice were initially perfused *in situ* with Hanks’ buffer ((#H9269; Sigma-Aldrich) through the opposite cannula for 2–3 min at a flow rate of 20 ml/min, which was followed by a collagenase perfusion with 150 ml of Hanks’ buffer supplemented with 2.5 mM of CaCl_2_ and 32 mg of collagenase (#C5138; Sigma-Aldrich). The PMH were dissociated after perfusion from the digested liver by gently scraping with a glass rod, suspended in DMEM/F-12 medium (GIBCO-BRL), and filtered through gauze. The cell suspension was then fractionated by Percoll density centrifugation (#GE17–5445-01; Sigma-Aldrich) at 2,500 rpm for 5 min at 4°C. The viability of isolated hepatocyte was assessed by trypan blue dye exclusion, and the cells used for the experiments had a viability greater than 88%. The isolated PMH were suspended in DMEM/F-12 medium containing 10% of fetal bovine serum, 1 nM of bovine insulin, 1% of penicillin-streptomycin, 50 nM of hydrocortisone (#H2270; Sigma-Aldrich), and 0.15 mg/ml of methionine (#M5308; Sigma-Aldrich), plated in culture dishes coated with 0.03% rat tail collagen (#A1048301; ThermoFisher), and cultured in a 5% CO_2_ atmosphere at 37 °C. After 3 h, the culture medium was changed to serum-free medium containing 1% of penicillin-streptomycin. PMH on the culture dish were treated with ethanol (85 mM) and the anti-HIV drugs the same as in AML12 or HepG2 cells. For cell death count, PMH were stained with Sytox green (1μM; Molecular Probes, Eugene, OR) and counted according to previously described methods [12].

### Molecular Anylases

After the treatments, a portion of the treated cells was washed with ice cold PBS and prepared for staining with Syntax green for cell death counting or immunohistochemistry described previously [21]. Other portions of the cells were detached with 0.05% Trypsin-EDTA solution for RNA isolation for real time PCR or for protein extraction for Western blotting. Total RNA was extracted using the RNeasy Mini Kit (Qiagen, Hilden, Germany, #74136) and reverse transcription was performed with QuantiTect Reverse Transcription Kit (Qiagen, #205311) according to the manufacturer’s instructions. For semiquantitative PCR, the KiCq Start One Step Probe RT-qPCR Ready mix or Ready Mix Taq PCR Kit (Sigma-Aldrich, # KCQS07 or D6442) was used. The PCR primers for p21 and igf1 were forward-CCTGGTGATGTCCGACCT G reverse-CCATGAGCGCATCGCAATC and forward-TAGAGCCTGCGCA ATGGAAT reverse-GGTGTGCATCTTCACCTTCA.

Primers for Usp10, Usp17 and Usp20 were forward-CTGCCATTC TGTCCCGTCTT reverse-CCACTGTATGGAGGAAGCTCA, forward-CGGTGCTCTTCTTTCTTACCCT reverse-GGCAAGTCGGTCTGTCTGTT, and forward-CCTCTGAGCATTGGCGACC and reverse-ACGACTGACAGGTTCCCTTAG, respectively. Primers for Rab18 and RCE1 were forward-GGCCATGTGGCAATACGATAC reverse-CACAGGTTTTTGCACTTGCCT, and forward-CTACGTCTGGAAGAGCGAACT reverse-CCCAGGAAAAGAATCATGGTCA. Primers for GAPDH were forward-CATGGGGAAGGTGAAGGTCG reverse-GTGATGGCATGGACTGTGGT. PCR cycling conditions were 94°C for 4 min followed by 35 cycles of 94°C for 10s, 63°C for 30s and 72°C for 30s, and 72°C for 8 min. The PCR products were quantified with the Delta Ct methods described before [13], or resolved on 2% agarose gels, stained with Safe DNA Gel Stain (APExBio, Boston, MA, cat. no. A8743) and visualized under ultraviolet illumination using Fusion image capture (PEQLAB Biotechnologies GmbH, Erlangen, Germany).

Extraction of proteins from the cell and liver tissues, immunohistochemistry and fluorescence immunohistochemistry were conducted according to the method described before [12, 13]. The Thermo Scientific RIPA Lysis and Extraction Buffer (#89900) or NE-PER Nuclear and Cytoplasmic Extraction Reagents (#78835) were used to lyse cells and extract proteins according to the manufacturer’s instructions. Antibodies for p21 (#ab109199), GRP75 (#ab227215), USP10 (#ab70895), USP20 (#ab72225), and RAB18 (#ab182764) were from Abcam. Antibodies for GRP78 (#3183) and senescence-associated β-galactosidase (SA-β-gal) and kit (#9860) were from Cell Signaling. SA-β-gal staining of the liver cells was conducted according to the manufacture’s protocol. Antibodies for IGF1 (#20214–1-AP) and PPARγ (#16643–1-AP) were from Proteintech Group, Inc. (Rosemont, IL, USA). Antibodies for CHOP (#sc-15204–1-AP) were from Santa Cruz. Antibodies for tubulin (#T6199) and GAPDH (#G8795) were from Sigma. Anti-bodies & kit for Ki-67 (#LC-C336576) were from LS Bio (Newark, CA) Quantitative immunoblot analysis was conducted with ImageJ (NIH) using GAPDH or tubulin as normalization controls.

### Statistical Analysis

All experiments were performed in triplicates. Data are presented as means ± SEM unless otherwise indicated. Statistical analyses were performed with GraphPad Prism^®^ 6 using the one way-ANOVA for comparison of multiple groups and t-test for comparison of two groups or treatments. The level of significance was set at p<0.05.

## Results

### 1. Cellular stresses in senescent cells exposed to antivirals

The status of senescence of the culture liver cell lines, HepG2 and AML-12, was induced by hydrogen peroxide (H_2_O_2_) treatments. The senescence associated β-galactosidase (SA-β-Gal) is a biomarker for cell senescence [22, 23]. *In situ* SA-β-Gal staining was more abundant in HepG2 and AML-12 cells treated with H_2_O_2_ compared to cells without the H_2_O_2_ treatment (Figure 1A). The status of senescence was further confirmed by measuring expression of cyclin-dependent kinase inhibitor CKI (p21) in the liver cells. Both the mRNA and protein of p21 were markedly increased in the senescent cells compared to non-senescent control HepG2 and AML-12 cells (Figure 1B-D). Insulin-like growth factor 1 (IGF1) is often associated with senescence of other experimental models including worms, flies, cultured cardiomyocytes, and skeletal muscle. However, neither the mRNA nor the protein expression of IGF1 was obviously changed in the senescent AML-12 cells compared to the corresponding control cells (Figure 1E-G). In HepG2 cells, IGF1 protein was not changed between senescence and non-senescence.

**Figure 2:**
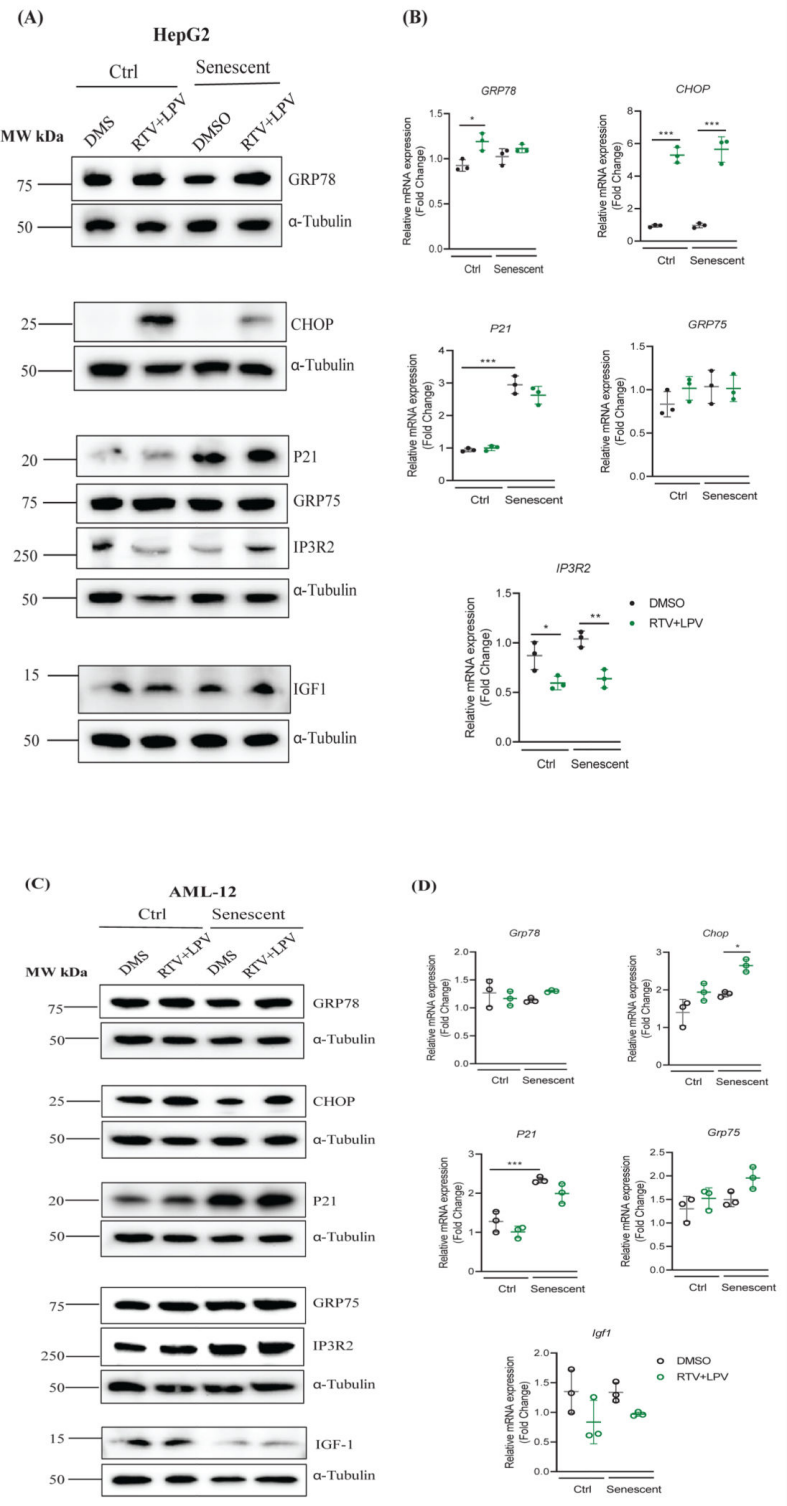
Cellular Stress Response in Non-senescent and Senescent Hepatocytes in Response to Anti-HIV Protease Inhibitor Treatments. (A) Western blots of selected stress response proteins in HepG2 treated with anti-HIV drugs; (B) RT-PCR of mRNA of stress response factors in HepG2 treated with anti-HIV drugs. (C) Western blots of stress response proteins in AML-12 treated with anti-HIV drugs; (D) RT-PCR of mRNA of stress response factors in AML-12 treated with anti-HIV drugs; DMS, dimethyl sulfoxide as vehicle control; RTV+LPV, ritonavir plus lopinavir; GRP78, glucose-regulated protein 78; CHOP, DNA damage-inducible transcript 3, also known as C/EBP homologous protein; P21, cyclin-dependent kinase inhibitor 1; GRP75, a member of the heat shock protein 70 gene family; IP3R2, the inositol 1,4,5-trisphosphate (IP3) receptor (IP3R) type 2; IGF1, insulin like growth factor 1; *, p<0.05; **, p<0.01; ***, p<0.005 compared to control cells or DMS vehicle.

Endoplasmic (ER) stress has been observed in non-senescent cell and non-aging animal models treated with anti-HIV protease inhibitors [12, 24]. To examine how endoplasmic (ER) stress responses were different in the senescent liver cells treated with anti-HIV drugs, we treated AML-12 and HepG2 senescent cells with the combination of ritonavir (RTV) and lopinavir (LPV) that mimics current regimen for HIV-infected patients. In HepG2 cells, compared between non-senescent and senescent cells, the basal protein but not mRNA of ER stress marker, GRP78 that regulates unfolded protein response, was suppressed by senescence, and senescence did not affect expression of protein or mRNA of another ER stress marker, CHOP that regulates ER stress-related cell death (Figure 2). Upon drug treatment with RTV and LPV, the mRNA and protein expression of GRP78 and CHOP was upregulated in either non-senescent control or senescent HepG2 cells, and the induction of CHOP protein was less dramatic in senescent HepG2 cells (Figure 2). Similarly, in AML-12 cells without the drug treatments, there was not significant difference between senescence and non-senescence in GRP78 expression, and senescence slightly suppressed CHOP expression (Figure 2 C&D). The treatment of RTV and LPV induced the mRNA and protein expression of CHOP in both control and senescent AML-12 cells and the induction was more significant in senescent than in non-senescent cells (Figure 2 C&D), suggesting that senescent liver cells are more sensitive to the ER stress-induced cell death.

To address the question whether the treatment of RTV and LPV could affect the process of senescence, several senescence-related factors were examined. The protein but not mRNA expression of P21 or IGF1 was upregulated after the treatment of RTV and LPV in both control and senescent liver cells (Figure 2). Notably, the upregulation of P21 protein levels after the treatment of RTV and LPV was much more remarkable in senescent HepG2 and AML-12 cells than in non-senescent control cells (Figure 2A&B). The heat shock protein GRP75 and the inositol 1,4,5-trisphosphate receptor type 2 (IP3R2) are two factors governing intracellular Ca^2+^ buffering [25], which were suggested recently to influence senescence and aging [26, 27]. In non-senescent control HepG2 cells, RTV and LPV upregulated the protein expression of GRP75 without altering its mRNA and suppressed either mRNA or protein expression of IP3R2 (Figure 2A&B). In senescent HepG2 cells, RTV and LPV slightly increased GRP75 protein without altering its mRNA expression. RTV and LPV significantly increased IP3R2 protein and suppressed IP3R2 mRNA. The anti-HIV drugs did not have an apparent impact on the expression of IGF1. In AML-12 cells, there was a trend that RTV and LPV increased both GRP75 protein and mRNA regardless the status of senescence. IP3R2 was increased in senescent AML-12 compared to non-senescent AML-12. IP3R2 was increased by the anti-HIV drugsin non-senescent AML-12 but not in senescent AML-12. IGF1 protein was reduced in the senescent AML-12 cells (Figure 2C) and IGF1 mRNA was not significantly changed in senescent or drug-treated AML-12 cells (Figure 2D).

**Figure 3:**
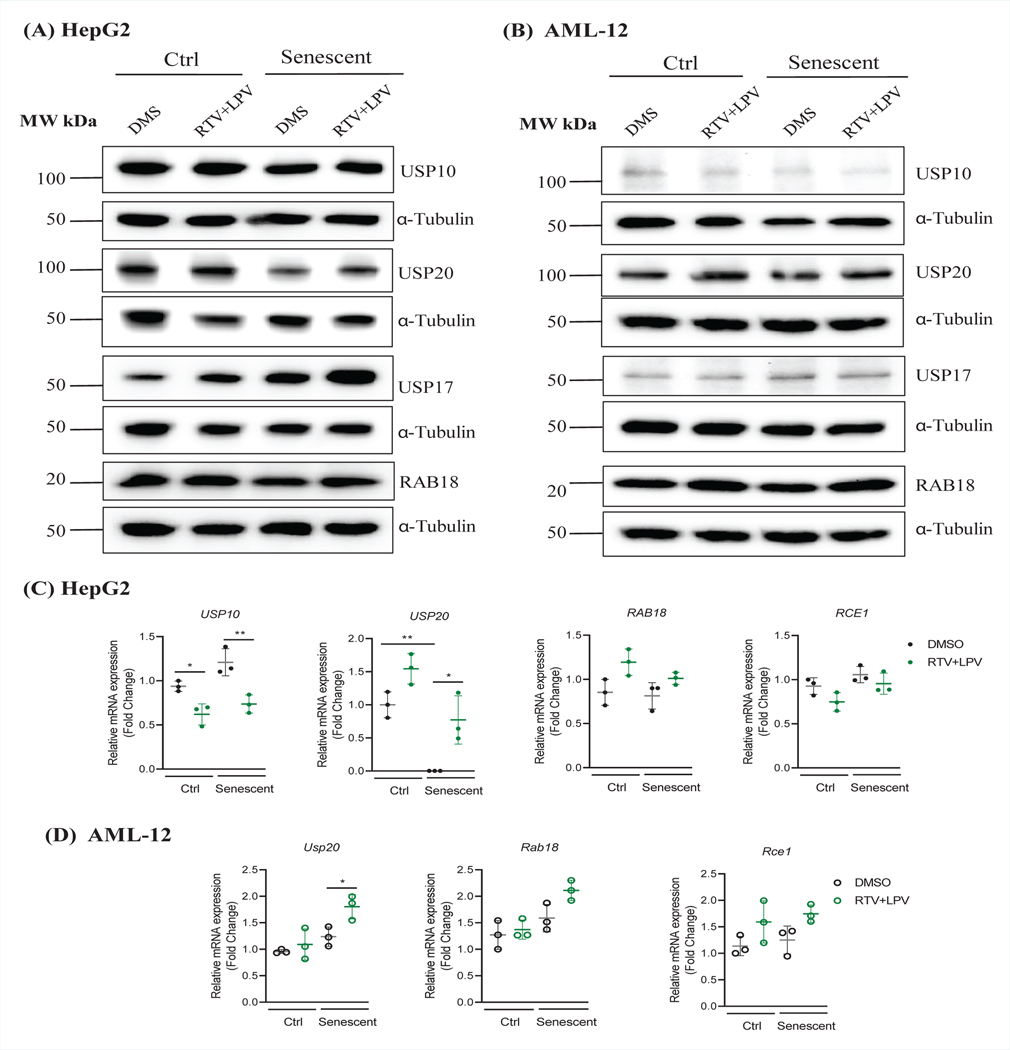
Changes of Cellular Stress-related Metabolic Proteins in Non-senescent and Senescent Hepatocytes in Response to Anti-HIV Protease Inhibitor Treatments. (A) Western blots of selected metabolic proteins in HepG2 treated with anti-HIV drugs; (B) Western blots of metabolic proteins in AML-12 treated with anti-HIV drugs; (C) Quantitation of metabolic proteins in HepG2; (D) Quantitation of metabolic proteins in AML-12; DMS, dimethyl sulfoxide as vehicle control; RTV+LPV, ritonavir plus lopinavir; USP10, deubiquitinase 10 involved in diverse cellular processes; USP20, ubiquitin specific peptidase 20; RAB18, a member of the Rab family of Ras-related small GTPases; USP17, ubiquitin-specific protease 17; RCE1, Ras converting enzyme 1; *, p<0.05; **, p<0.01; ***, p<0.005 compared to control cells or DMS.

Other factors identified previously by us to be relevant to anti-HIV drug hepatoxicity were also examined in the senescent liver cells [8]. In HepG2, senescence slightly reduced the protein levels of ubiquitin-specific protease 10 (USP10) and there was an overall moderate increase of USP10 mRNA in senescent cells (Figure 3A&C). Treatment of RTV and LPV had no apparent effects on USP10 protein but reduced mRNA of USP10 more in senescent than in non-senescent cells. Senescence reduced both protein and mRNA levels of ubiquitin-specific protease 20 (USP20) significantly (Figure 3A&C). Upon the anti-HIV drug treatment, both the mRNA and protein expression of USP20 was upregulated and the upregulation was stronger in no-senescent than in senescent cells (Figure 3A&C). Senescence significantly increased protein levels of ubiquitin-specific protease 17 (USP17), which was further increased by the RTV and LPV treatments (Figure 3A). RAB18 is a member of the Rab family of small GTPases that involve in ER-Golgi trafficking, organellar stress response, and lipid accumulation in the liver [8]. There were trends of increase of RAB18 and decrease of Ras converting enzyme 1 (RCE1) in response to RTV and LPV, which were not affected by the status of senescence of HepG2 (Figure 3A&C). In AML-12 cells, the protein expression of USP10 was also suppressed whereas the protein expression of USP20 was slightly upregulated in senescent cells compared to non-senescent control cells (Figure 3B&D). The treatments of RTV and LPV suppressed the protein expression of USP10 but upregulated the protein expression of USP20 in both non-senescent and senescent AML-12 cells. The increase of USP20 mRNA by RTV and LPV was significant only in senescent AML-12 cells (Figure 3D). Interestingly, USP17 was increased in senescent AML-12 regardless of the drug treatment. RAB18 protein was upregulated in eithernon-senescent control or senescent AML-12 cells in response to RTV and LPV treatments (Figure 3B). However, the anti-HIV drugs did not affect mRNA expression of Rab18 or Rce1 significantly in AML-12 cells (Figure 3D).

### 2. Cellular stresses in senescent AML-12 exposed to hippuric acid and antivirals

**Figure 4:**
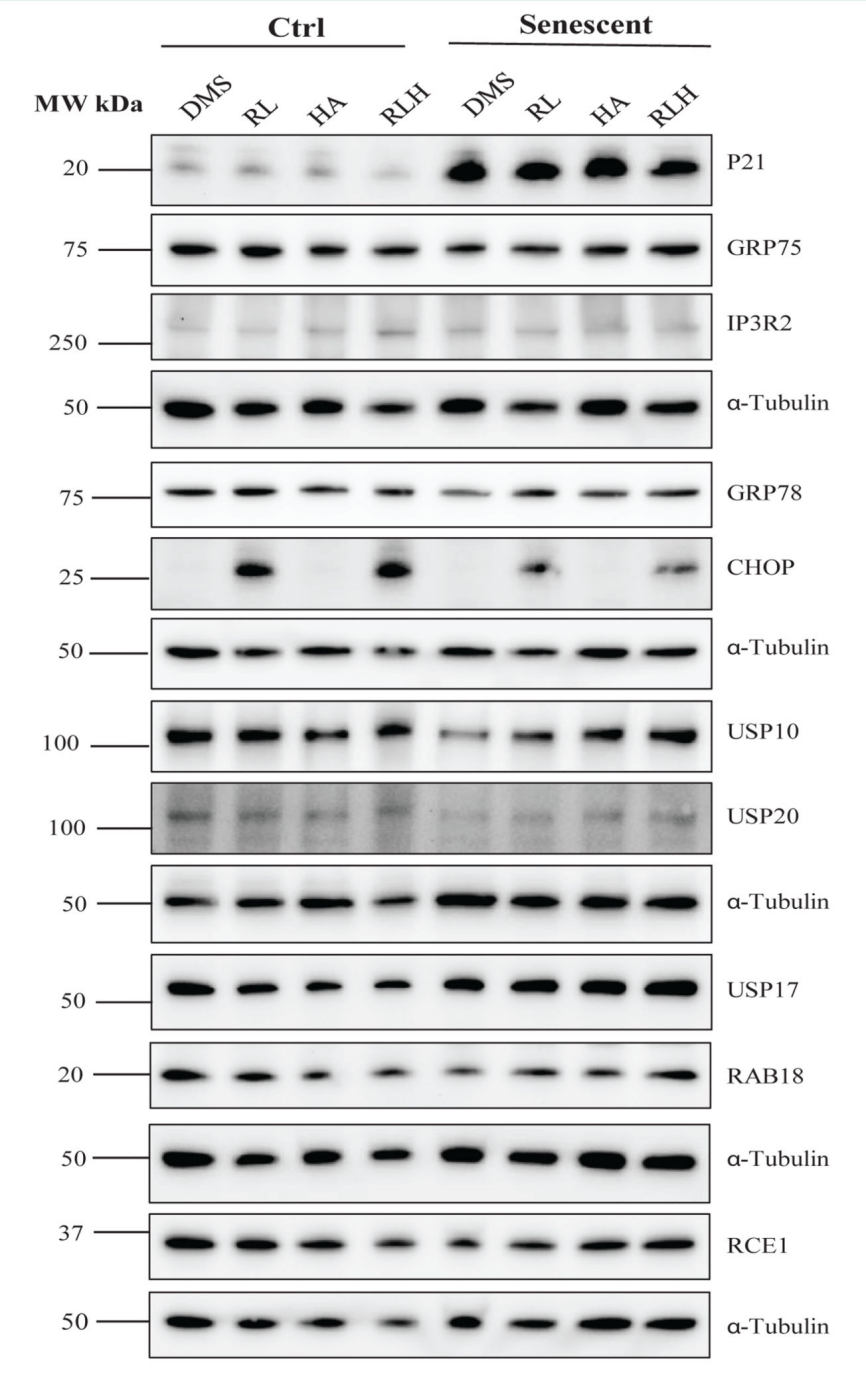
Cellular Stress Response in Non-senescent and Senescent AML-12 in Response to Anti-HIV Drug and Cocaine Derivative Treatments. DMS, dimethylsulfoxide as vehicle control; RL, ritonavir plus lopinavir; HA, hippuric acid (a cocaine derivative); RLH, ritonavir plus lopinavir plus hippuric acid; Western blots of selected metabolic proteins include: P21,cyclin-dependent kinase inhibitor 1; GRP75, a member of the heat shock protein 70 gene family; IP3R2, the inositol 1,4,5-trisphosphate (IP3) receptor (IP3R) type 2; GRP78, glucose-regulated protein 78; CHOP, DNA damage-inducible transcript 3, also known as C/EBP homologous protein; USP10, deubiquitinase 10 involved in diverse cellular processes; USP17, ubiquitin-specific protease 17; USP20, ubiquitin specific peptidase 20; RAB18, a member of the Rab family of Ras-related small GTPases; RCE1, Ras converting enzyme 1.

Anti-HIV drug use with cocaine abuse occurs in some HIV-infected patients, which contributes to significant neurological disorders [5, 6, 28]. We were interested to evaluate the hepatotoxic effects of the anti-HIV drugs combined with cocaine in AML-12 cells. Because cocaine is metabolized to hippuric acid in the liver [29, 30], we treated the cells with hippuric acid instead of cocaine which is a controlled substance with limited access. Hippuric acid alone had no effects on the expression of P21 or GRP75 in either non-senescent or senescent AML-12 cells (Figure 4) and supplemental Fig. S1A). Combination of hippuric acid with ritonavir and lopinavir did not further increase or decrease the increased expression of P21 by the anti-HIV drugs in the senescent cells. Hippuric acid alone slightly increased IP3R2 in both control and senescent cells and additive effects on IP3R2 were observed in control but not in senescent AML-12 cells treated with the combination of hippuric acid and the HIV drugs. The ER stress response markers of GRP78 and CHOP were induced by ritonavir and lopinavir stronger in non-senescent than in senescent AML-12 cells, and the expression of GRP78 and CHOP was additively increased by hippuric acid combined with the two HIV drugs (Figure 4 and Figure S1B). Similar to ritonavir and lopinavir, hippuric acid reduced the expression USP10 and USP20 regardless of the status of senescence of AML-12 (Figure 4 and Figure S1C). In the non-senescent cells, hippuric acid had no effects on the expression of USP17 but decreased the USP17 expression that was increased by ritonavir and lopinavir. In the senescent cells, hippuric acid alone increased USP17 expression but did not exert additive effects on USP17 when combined with the two HIV drugs. RAB18 was reduced by hippuric acid in either senescent or non-senescent AML-12. However, in combination with HIV drugs, hippuric acid increased the expression of RAB18 in the senescent cells (Figure 4 and Figure S1D). In addition, Hippuric acid alone or in the drug combination had no effects on the expression of RCE1 which was reduced by the anti-HIV drugs in both senescent and non-senescent cells.

### 3. Cellular stresses in senescent HepG2 exposed to alcohol and hippuric acid

**Figure 5:**
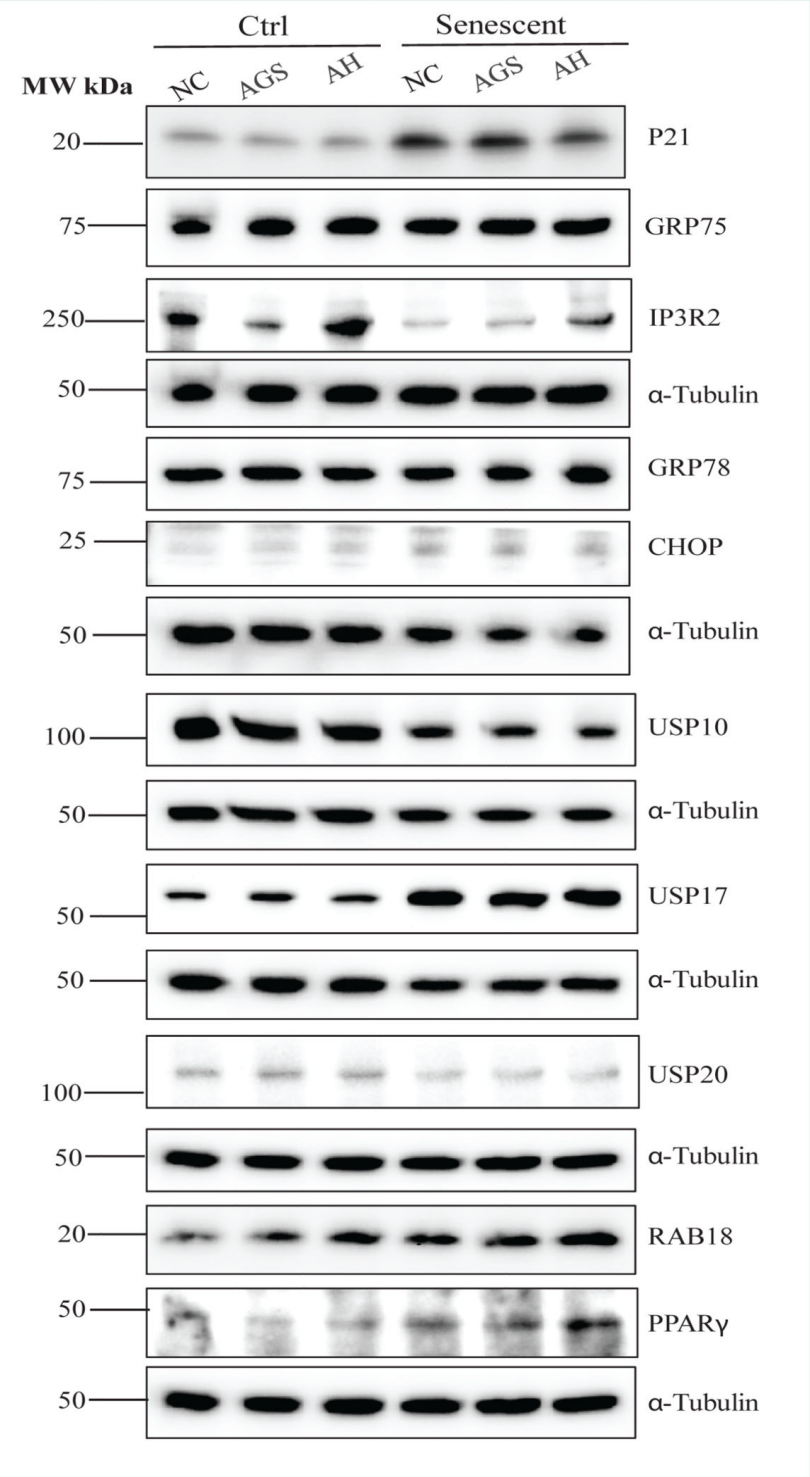
Cellular Stress Response in Non-senescent and Senescent HepG2 in Response to Ethanol and Cocaine Derivative Treatments. NC, control without drug or ethanol; AGS; cell incubated in an acetaldehyde generating system/medium consisting of ethanol, alcohol dehydrogenase (ADH) and nicotinamide adenine dinucleotide (NAD+); HA, hippuric acid (a cocaine derivative in the liver); AH, AGS plus hippuric acid; Western blots of selected metabolic proteins include: P21, cyclin-dependent kinase inhibitor 1; GRP75, a member of the heat shock protein 70 gene family; IP3R2, the inositol 1,4,5-trisphosphate (IP3) receptor (IP3R) type 2; IGF1, insulin like growth factor 1; GRP78, glucose-regulated protein 78; CHOP, DNA damage-inducible transcript 3, also known as C/EBP homologous protein; USP10, deubiquitinase 10 involved in diverse cellular processes; USP20, ubiquitin specific peptidase 20; RAB18, a member of the Rab family of Ras-related small GTPases; PPARγ, peroxisome proliferator-activated receptor γ; USP17, ubiquitin-specific protease 17.

HepG2 cells metabolize alcohol little due to low expressing of cytochrome P-450 2E1 (CYP2E1) or alcohol dehydrogenase [31]. To investigate alcohol effects on HepG2, we incubated the liver cells in an acetaldehyde generating medium/system (AGS) consisting of alcohol, alcohol dehydrogenase (ADH) and nicotinamide adenine dinucleotide (NAD+) [19]. In control non-senescent HepG2 cells, the expression of P21 was suppressed by alcohol treatment (AGS alone) and stayed unchanged after the treatment of the combination of alcohol and hippuric acid (Figure 5 and Figure S2). In contrast, in senescent HepG2 cells, the treatment of alcohol alone did not alter the expression of P21 but the combination of alcohol and hippuric acid suppressed the expression of P21. In the control HepG2 cells, alcohol alone obviously suppressed the expression of IP3R2 whereas the addition of hippuric acid to AGS brought the level of IP3R2 back to normal. In contrast in the senescent HepG2, the expression of IP3R2 was reduced compared to the non-senescent control, which was increased after the treatment of alcohol and was further increased by the addition of hippuric acid, indicative of additive effects of drug and hippuric acid only in senescent HepG2 cells. Neither alcohol alone or alcohol in combination with hippuric acid affected the expression of GRP75 in the control or senescent HepG2 cells (Figure 5 and Figure S2A). The expression of ER stress markers of GRP78 and CHOP was upregulated after the treatment of alcohol alone or in combination with hippuric acid in both control and senescent HepG2 cells (Figure 5 and Figure S2B). However, the additive effects of alcohol and hippuric acid on the stimulation of ER stress were more obvious in senescent HepG2 cells than the control non-senescent cells. There were trends of reduction of USP10 or USP20 and increase of USP17 in the control HepG2 cells in response to alcohol (Figure 5 and Figure S2C). Hippuric acid did not have any effects on the expression of USP10, USP17, or USP20 in the control cells. In the senescent HepG2, the reduction of USP10 and USP20 by alcohol was further reduced which was not affected by addition of hippuric acid. However, USP17 was further increased by hippuric acid in the senescent cells. PPARγ (Peroxisome proliferator-activated receptor γ) acts as a key mediator of lipid metabolism, which may influence cellular senescence [32]. The alcohol treatment increased the expression of PPARγ as well as RAB18 slightly in the non-senescent control cells and markedly in the senescent HepG2 cells, the latter of which was further increased by the addition of hippuric acid, indicative of synergistic effects of alcohol and hippuric acid on these two factors in the senescent cells (Figure 5 and Figure S2D).

### 4. Stress and injury in senescent hepatocytes and aged liver of mice fed alcohol and/or antivirals

**Figure 6:**
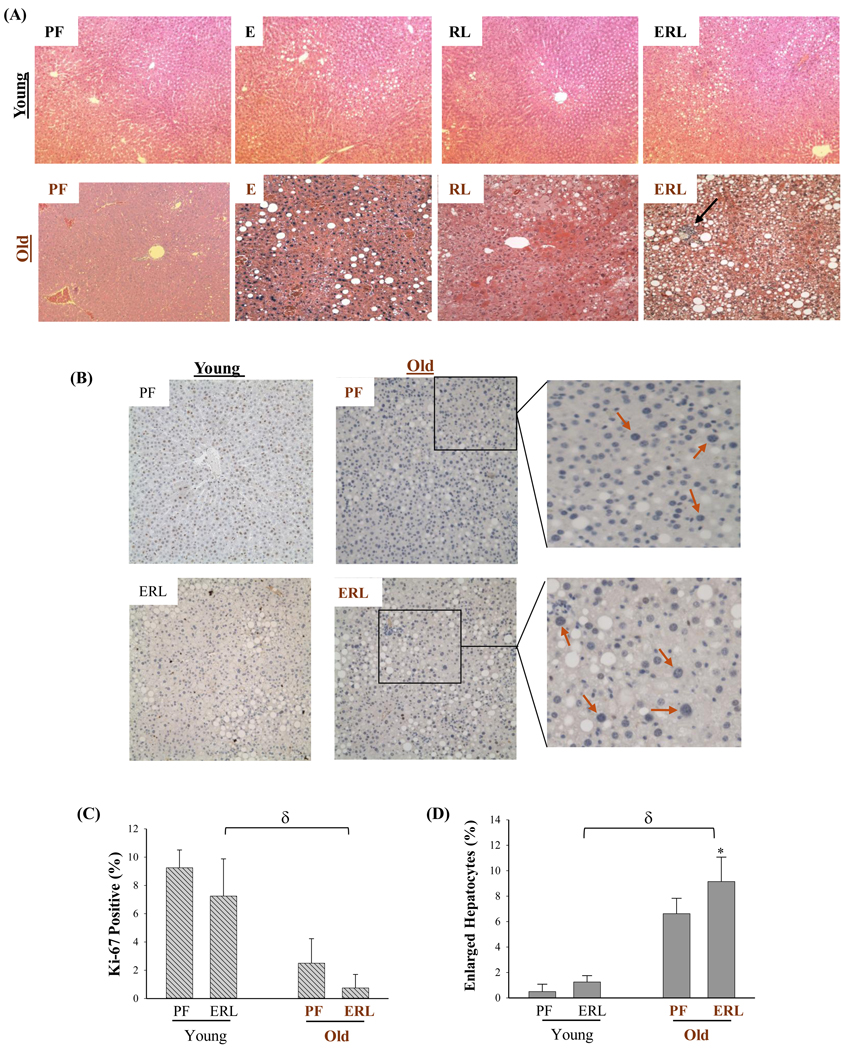
Fatty Liver Injury in Young and Old Mice Treated with Ethanol and Anti-HIV Protease Inhibitors. (A) H&E staining of liver tissues of mice treated with ethanol and/or anti-HIV drugs; Blue arrow identifies neutrophil infiltration and necrotic foci. (B) Immunohistochemistry of liver tissues with anti-Ki-67 antibodies; Red arrows highlight markedly enlarged hepatocytes in old mice. (C) Quantitation of Ki-67 positive liver cells compared between young and old mice. (D) Quantitation of enlarged hepatocytes compared between young and old mice; PF, pair-fed control; E, fed with ethanol; RL, treated with ritonavir and lopinavir; ERL, fed ethanol and treated with ritonavir and lopinavir; *, p<0.05 compared to PF in the same animal group;^δ^, p<0.01 compared between young and old mice.

**Figure 7:**
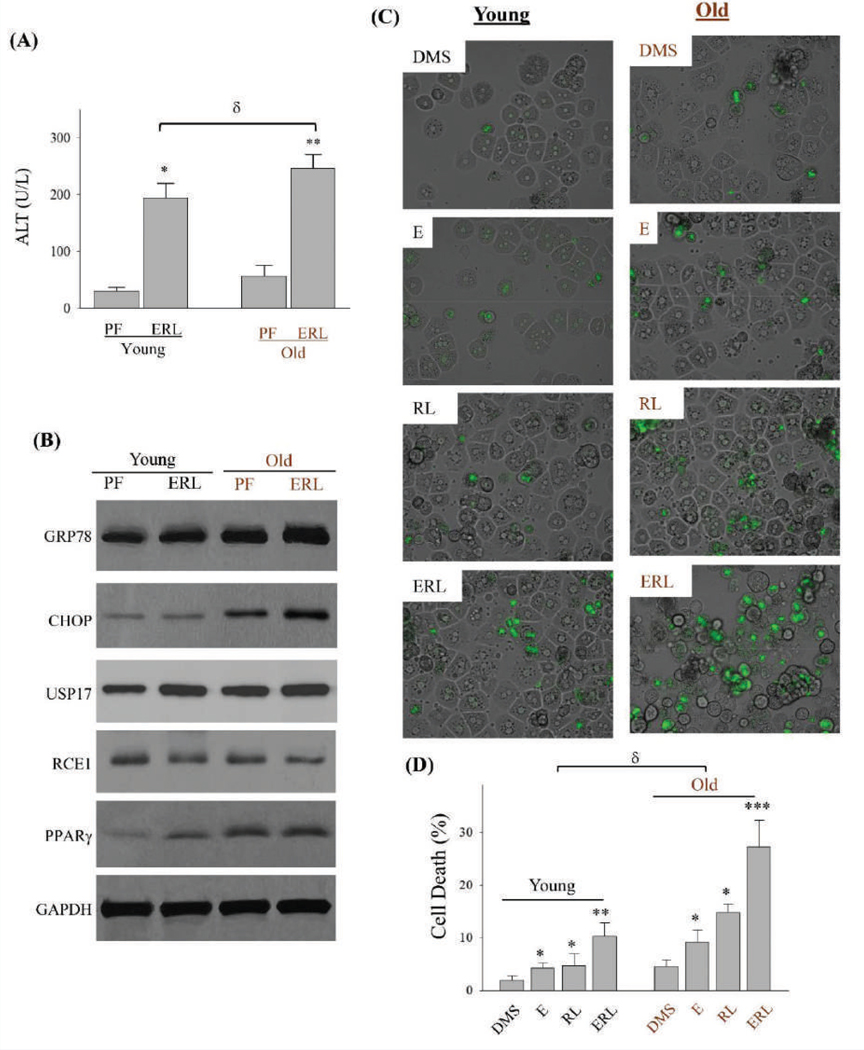
Serum Levels of Alanine Amino transfer as e and Hepatic Stress and Injury in Young and Old Mice Treated with Ethanol and Anti-HIV drugs. (A) Serum levels of alanine aminotransferase (ALT); PF, pair-fed control diet; ERL, fed with ethanol diet, ritonavir and lopinavir. (B) Western blots of selected stress marker proteins from liver of mice; GRP78, glucose-regulated protein 78; CHOP, DNA damage-inducible transcript 3, also known as C/EBP homologous protein; USP17, ubiquitin-specific protease 17; RCE1, Ras converting enzyme 1; PPARγ, peroxisome proliferator-activated receptor γ. (C) Death of primary hepatocytes isolated from livers of young or old mice. The liver cells were treated with ethanol, ritonavir and lopinavir and then stained with SYTOX Green. Green-fluorescent nuclei indicate cell death; (D) Quantitation of cell death; DMS, dimethyl sulfoxide as vehicle control; E, ethanol; RL, ritonavir plus lopinavir; ERL, ethanol plus ritonavir and lopinavir; *, p<0.05; **, p<0.01; ***, p<0.005 compared to PF or DMS in the same age group. ^δ^, p<0.05 compared between young and old.

As hippuric acid yielded mixed effects on AML-12 and HepG2, the investigation was extended into *in vivo* animal models treated with alcohol and the anti-HIV drug. Both young mice (two months old) and old mice (23 months old) had fat accumulations in the liver after being fed with alcohol diet for two months (Figure 6A). Compared to alcohol diet feeding, treatment of the anti-HIV drugs induced less fat accumulation in either young or old mice. Hepatic triglycerides were significantly increased in the alcohol treated young mice and further increased in the alcohol treated old mice (Figure S3). The anti-HIV drugs increased the triglycerides lightly in young mice and significantly in the old mice. More foamy and swelled hepatocytes were observed in old mice than in young mice in response to the HIV drug treatment. Combination of the HIV drugs with alcohol induced much severer fatty liver and inflammation in the old mice than in young mice. Of note, in the liver of old mice treated with alcohol and the HIV drugs, Ki-67 positive cells were significantly lower in the liver of old mice than in young mice (Figure 6 B&C). Interestingly, marked increase of enlarged nuclei of undividing hepatocytes were observed in old mice compared to young mice, and there was a significant increase in the number of enlarged nuclei in alcohol and HIV drug treated old mice compared to pair-fed control (Figure 6 B&D), indicative of accelerated senescence/aging of the liver. Serum levels of alanine aminotransferase (ALT) indicating liver injury were markedly increased in both young and old mice treated with alcohol and the HIV drugs (Figure 7A), and the ALT levels were significantly higher in old mice than in young mice with the same alcohol and drug treatments. The drug and alcohol also induced cellular stress in the mouse liver. ER stress as indicated by increased expression of GRP78 was observed in the liver of both young and old mice. Compared to young mice, old mice had even higher expression of GRP78 and CHOP (Figure 7B) and (Table S1). USP17 and PPARγ were upregulated in young mice in response to alcohol and drug treatments, which were increased more in old mice than in young mice. In response to treatments of the HIV drug and alcohol, there was a trend of reduction of RCE1 expression in young mice and an apparent reduction of RCE1 in old mice. Further, the drug and alcohol induced cell death in primary hepatocytes isolated from the livers of young and old mice (Figure 7C). The drug and alcohol combination induced much severer death of the hepatocytes from old mouse liver than from young mouse liver (Figure 7 C&D). The death rate of old liver cells could reach 29% when treated with alcohol and the HIV drugs.

## Discussion

The prevalence of age-related liver pathologies has been found to be considerably higher in people with HIV than in people without HIV, which could be due partly to cellular stress and senescence and consequent inflammation. Knowledge is limited on whether alcohol, anti-HIV ritonavir, lopinavir and other substance use disorders have impact on senescence of hepatocytes and aging of liver and how alcohol and drug-induced cellular stresses are different in senescent liver cells or aging liver. In this study, we investigated for the first time these potential effects using cultured liver cell lines and animal models and made a few interesting findings. First, we applied two liver cell lines, HepG2 and AML-12. HepG2 cells are derivatives of human liver cancer cells and AML-12 are derivatives of normal mouse liver cells with epithelial morphology. Although there were differences in the expression of individual stress proteins/factors in response to alcohol or HIV drugs, senescence could be induced in either HepG2 or AML-12 by hydrogen peroxide (H_2_O_2_) acting as reactive oxygen species (ROS) promoting cellular stress responses. In line with the H_2_O_2_ as a reliable senescence inducer, either alcohol or the anti-HIV drugs are known to generate various kinds of stress responses including the ER stress and oxidative stresses [33–35], contributing to cell senescence. There are a few golden standards for detecting the senescence. In our hands, the senescence associated β-galactosidase (SA-β-Gal) and cyclin-dependent kinase inhibitor 1(P21) could be used to reliably detecting the liver cell senescence. Consistent results were not obtained when insulin-like growth factor 1 (IGF1) was used as the senescence marker for the liver cells despite IGF1 is often associated with senescence of cell/organ models other than the liver[33].

Second, there is no direct evidence from this study that the use of alcohol and/or anti-HIV drugs cause the senescence of hepatocytes. Unlike with hydrogen peroxide, exposing normal hepatocytes with anti-HIV drugs did not result in positive detections of SA-β-Gal or P21 (data not showed). Nevertheless, pieces of indirect evidence were obtained from this study indicating negative influence of alcohol and the HIV drugs on the liver cells once their senescence were primed by hydrogen peroxide. For instance, the ER stress indicators, GRP78 and CHOP, were found to be more sensitive to alcohol and the HIV-drugs in senescent hepatocytes or in old liver than in non-senescent hepatocytes or young liver, which are associated with increased non-dividing hepatocytes and cell death.

GRP75 and the ER resident IP3R2 are two factors that regulate and balance levels of intracellular Ca^2+^ that directly impact senescence or aging [36]. In the senescent liver cells, GRP75 protein tended to be unchanged whereas IP3R2 protein tended to be increased in response to the alcohol and drug treatment. This could break the balance of intracellular Ca^2+^ and promote senescence or aging. USP10 has been reported to promote SIRT6 degradation suppressing expression of lipogenic factors and reducing fat accumulation in the liver [37, 38] whereas USP20 has been reported to upregulate PPARγ promoting cell senescence via fat accumulation in the cells [32, 39, 40]. We found that, in the senescent hepatocytes, USP10 was inhibited and USP20 was increased by the alcohol and HIV drug treatments. Our results support the effects of the USP proteins on metabolic disorders and lipid accumulation in the liver [41]. With respect to USP17 that plays a critical role in regulating protein stability and cellular signaling pathways involving the Ras small GTPases family [42,43], we found that the protein expression of USP17 was upregulated in non-senescent HepG2 and AML-12 cells and increased further in senescent HepG2 and AML-12 cells in response to the anti-HIV drugs, which was associated with reduced RCE1 and increased RAB18. In the liver of old mice fed alcohol and the HIV drugs, USP17 was also markedly upregulated, which was associated with reduced RCE1 and increased PPARγ. Since RCE1 is known to be an off target of the anti-HIV protease inhibitors [14] and USP17 deubiquitinates RCE1[42], we believe that the anti-HIV drugs might induce organelle stress, mechanistically upregulate USP17 and subsequently reduce RCE1, which exacerbates RAB18 or PPARγ liver lipid accumulation contributing to liver senescence [44, 45]. In addition, the increase in ubiquitin-related markers (USP10 mRNA, USP17, USP20) in senescent cells suggests that ubiquitination could be regarded as a time-related marker associated with cell degradation, senescence or tumor progression in the liver, which also occurs in aging brain, adipose tissue, and skeletal muscle [46–52].

Third, with respect to the effects of hippuric acid on senescence, stress proteins and injury of hepatocytes, we found differential effects in terms of cell types or combinations with drugs and alcohol. Hippuric acid alone did not have significant effects on the expression of P21, GRP75, or IP3R2 in AML-12 cells, suggesting that the cocaine derivative alone might not affect the senescence of the cells being derived from normal mouse liver. Hippuric acid had some additive or negative effects on the expression of GRP78, CHOP, USP10 and RAB18 in AML-12 cell treated with the anti-HIV drugs. However, USP20, RCE1 and PPARγ were basically not changed in the cells treated with hippuric acid and the HIV drugs, suggesting that hippuric acid may induce some degree of cellular stress which might not severe enough to cause downstream cell injury in AML-12. In the liver cancer derived HepG2 cells, combination of hippuric acid and alcohol suppressed the expression of P21 and the senescence promoting factor IP3R2 but additively increased the expression of GRP78 and CHOP. The combination of hippuric acid and alcohol did not affect the expression of USP10, USP17 or USP20 but had synergistic effects on RAB18 and PPARγ, suggesting that hippuric acid and alcohol might have dual effects on the cancerous liver cells: protect against senescence but promote cellular stress. We speculate that the intriguing anti-senescence effects by hippuric acid might not occur *in vivo* considering that there are report that alcohol could boost cocaine’s bioavailability and alcohol combined with cocaine increased liver damage in rats [53, 54]. More experimental tests are needed to validate further the hepatotoxic effects by hippuric acid and alcohol.

**In summary**, in senescent HepG2 and AML-12 liver cells and in aged mouse liver treated with alcohol combined with anti-HIV ritonavir and lopinavir, ameliorating protein factors and responses that potentially function in reducing cellular stresses are impaired and detrimental factors that potentially promote cell dysfunction and death are exacerbated. Therapy targeting hepatic cellular stress might be effective to prevent liver injury in old people with HIV and drug/alcohol use disorders.

## Supplementary Material

Supplemental_Table_Figures-JAP_13-0059

## Figures and Tables

**Figure 1A-D:**
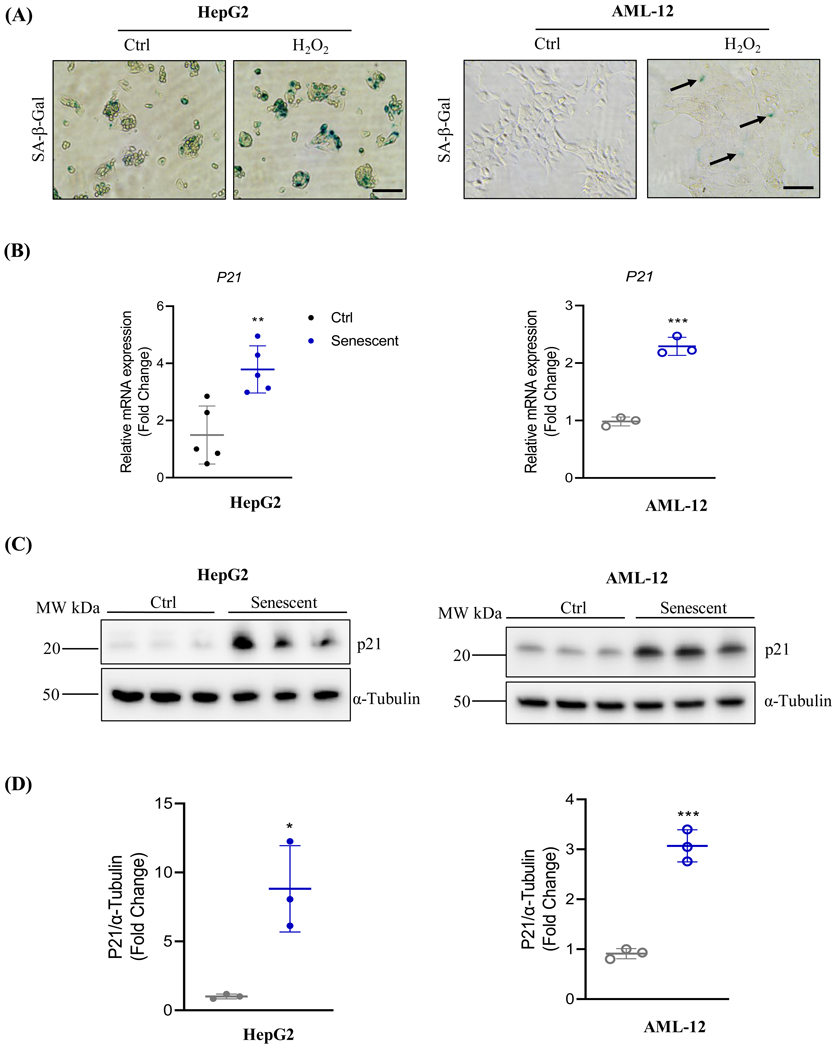
Induction and Detection of Senescence of Hepatocytes with Hydroperoxide. (A) Detection of Hydroperoxide (H_2_O_2_)-induced senescence of HepG2 or AML-12 with in-situactivity detection of senescence-associated β-galactosidase (SA-β-gal). Ctrl, control cells without H_2_O_2_ treatment. Blue color indicates SA-β-gal positive; (B) RT-PCR of mRNA of the senescence marker, p21 (cyclin-dependent kinase inhibitor 1) in the hepatocytes (C) Western blotting of p21 protein in the hepatocytes with tubulin as a loading control; (D) Quantitation of P21 protein expression in HepG2 or AML-12

**Figure 1E-G:**
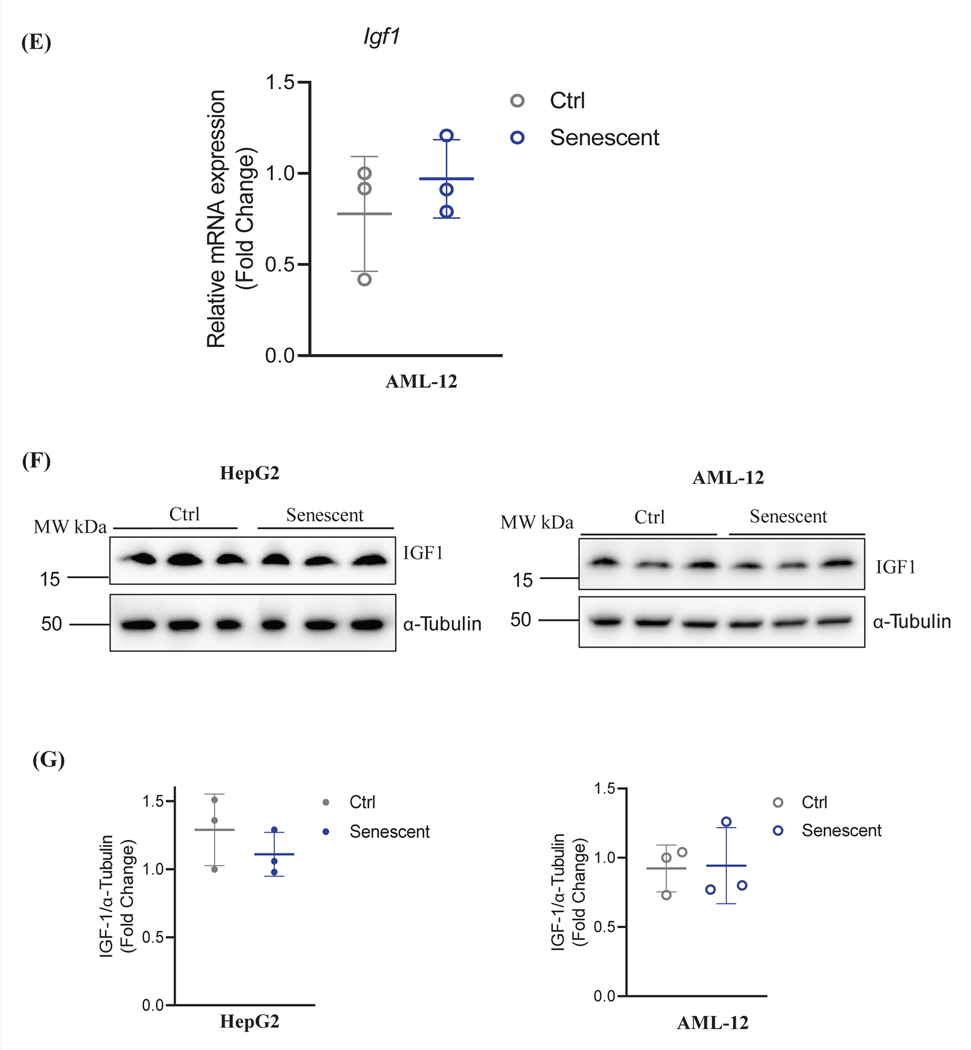
(E) RT-PCR of mRNA of igf-1 gene (insulin like growth factor 1) in AML-12; (F) Western blotting of IGF1 protein in HepG2 or AML-12; (G) Quantitation of IGF1 protein expression in the hepatocytes; *, p<0.05; **, p<0.01; ***, p<0.005 compared to control cells.
