# Supplementary material for: Alcohol, Anti-HIV Drugs, and/or Hippuric Acid Deteriorate Cellular Stresses in Senescent Hepatocytes and Aging Murine Liver
Source: J Addict Prev. Author manuscript; Available in PMC 2025 Sep 1. (PMC12129443; doi:10.13188/2330-2178.1000059)
Supplement: Supplemental_Table_Figures-JAP_13-0059 [file NIHMS2074340-supplement-Supplemental_Table_Figures-JAP_13-0059.pdf]

Supplemental Table S1

Table S1 Densitometric analyses of Western blot bands of selected stress marker proteins from liver of mice fed alcohol and/or anti-HIV drugs.

Young Mice											
	GRP78	Divide by GAPDH	CHOP	Divide by GAPDH	USP17	Divide by GAPDH	RCE1	Divide by GAPDH	PPARg	Divide by GAPDH	GAPDH
PF1	10212.02	0.84	1736.43	0.14	7312.38	0.60	9677.59	0.80	1915.38	0.16	12086.95
PF2	10354.44	0.86	1836.43	0.15	6991.72	0.58	9971.54	0.83	1994.50	0.17	11994.54
PF3	10571.37	0.88	1590.89	0.13	7060.13	0.59	10155.02	0.84	1885.21	0.16	12022.95
Mean		0.86		0.14		0.59		0.83		0.16	
SEM		0.010		0.006		0.007		0.013		0.003	
ERL1	12799.63	1.07	1948.55	0.16	10914.57	0.91	6908.35	0.58	4685.23	0.39	11973.54
ERL2	13458.05	1.13	2035.38	0.17	9553.98	0.80	6782.76	0.57	4948.05	0.42	11895.54
ERL3	12733.15	1.07	2012.38	0.17	11126.98	0.93	7242.42	0.61	5071.05	0.42	11947.13
Mean		1.09		0.17		0.88		0.58		0.41	
SEM		0.021		0.002		0.040		0.011		0.010	
P value vs. PF		0.00066		0.01980		0.00199		0.00146		0.00175	
Old Mice											
PF1	12154.13	1.02	4571.67	0.38	9838.57	0.82	8568.76	0.72	7153.37	0.60	11943.59
PF2	12493.02	1.04	4852.79	0.41	9279.21	0.78	8998.59	0.75	7264.95	0.61	11955.83
PF3	13095.88	1.09	4739.08	0.40	9090.50	0.76	8969.00	0.75	7697.20	0.64	11968.59
Mean		1.05		0.39		0.79		0.74		0.62	
SEM		0.022		0.007		0.019		0.011		0.014	
P value vs. Young		0.00150		0.00024		0.00672		0.00757		0.00504	
ERL1	14430.37	1.22	6934.88	0.59	11770.10	1.00	5869.45	0.50	8035.05	0.68	11801.81
ERL2	15035.66	1.26	7116.30	0.60	11216.69	0.94	5682.15	0.48	7936.88	0.67	11893.40
ERL3	13407.54	1.12	7241.00	0.61	10458.28	0.87	5522.74	0.46	8218.71	0.69	11954.81
Mean		1.20		0.60		0.94		0.48		0.68	
SEM		0.042		0.005		0.057		0.010		0.006	
P value vs. PF		0.03481		0.00186		0.03120		0.00068		0.0137	
P value vs. Young		0.00470		0.00020		0.0442		0.00219		0.00206	

Protein band density was analyzed with *ImageJ* and normalized with GAPDH (glyceraldehyde 3-phosphate dehydrogenase) in the same protein sample. PF, pair-fed control diet; ERL, fed with ethanol diet, ritonavir and lopinavir; GRP78, glucose-regulated protein 78; CHOP, DNA damage-inducible transcript 3, also known as C/EBP homologous protein; USP17, ubiquitin-specific protease 17; RCE1, Ras converting enzyme 1; PPARγ, peroxisome proliferator-activated receptor γ.

Supplemental Figure S1

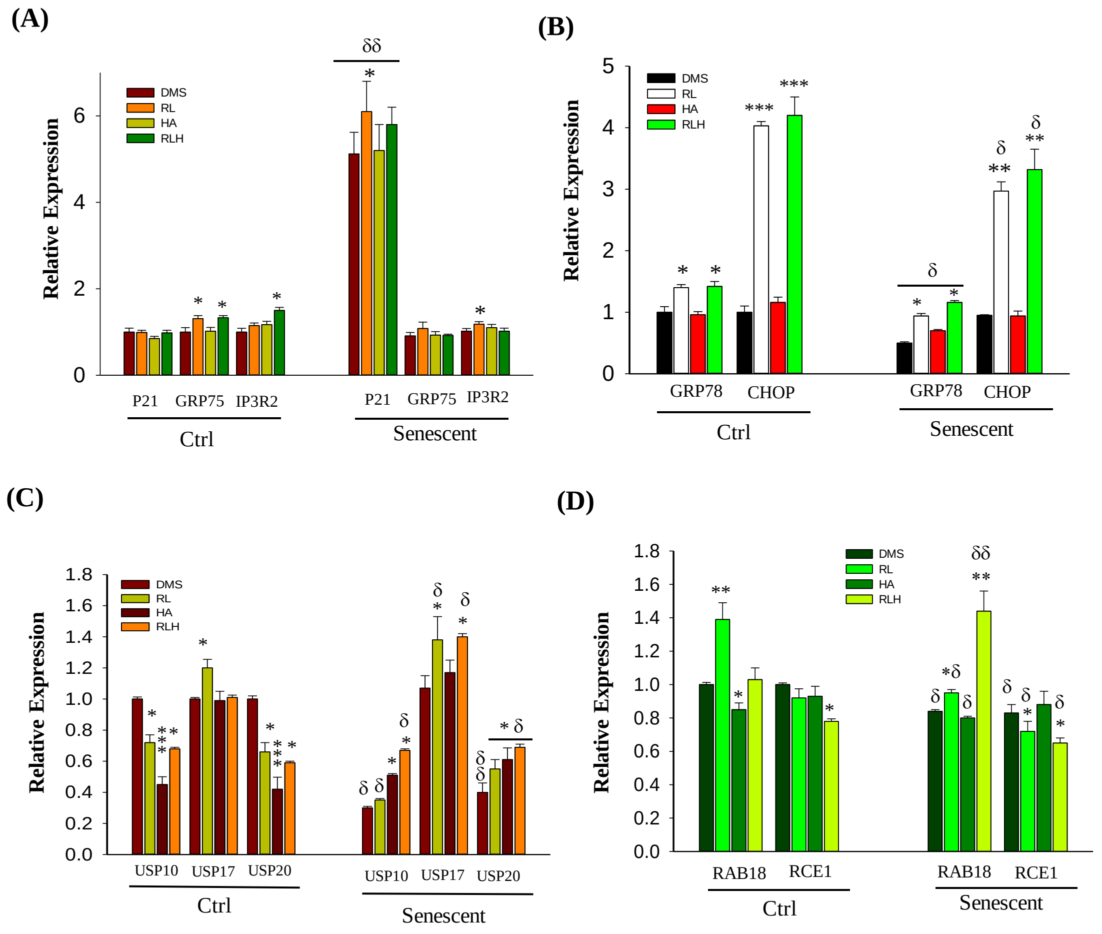

Figure S1 Cellular Stress Response in Non-senescent and Senescent AML-12 in Response to Anti-HIV Drug and Cocaine Derivative Treatments. Quantitation of protein expression normalized with corresponding loading controls of tubulin and relative protein expression of non-senescent cells treated with vehicle was set as one; DMS, dimethylsulfoxide as the vehicle control; RL, ritonavir plus lopinavir; HA, hippuric acid (a cocaine derivative); RLH, ritonavir plus lopinavir plus hippuric acid; Western blots of selected metabolic proteins include: (**A**) P21, cyclin-dependent kinase inhibitor 1; GRP75, a member of the heat shock protein 70 gene family; IP3R2, the inositol 1,4,5-trisphosphate (IP3) receptor (IP3R) type 2; (**B**) GRP78, glucose-regulated protein 78; CHOP, DNA damage-inducible transcript 3, also known as C/EBP homologous protein; (**C**) USP10, deubiquitinase 10 involved in diverse cellular processes; USP17, ubiquitin-specific protease 17; USP20, ubiquitin specific peptidase 20; (**D**) RAB18, a member of the Rab family of Ras-related small GTPases; RCE1, Ras converting enzyme 1. *, $p<0.05$; **, $p<0.01$; ***, $p<0.005$ compared to DMS; δ, $p<0.05$ and δδ, $p<0.01$ compared to control non-senescent cells.

Supplemental Figure S2

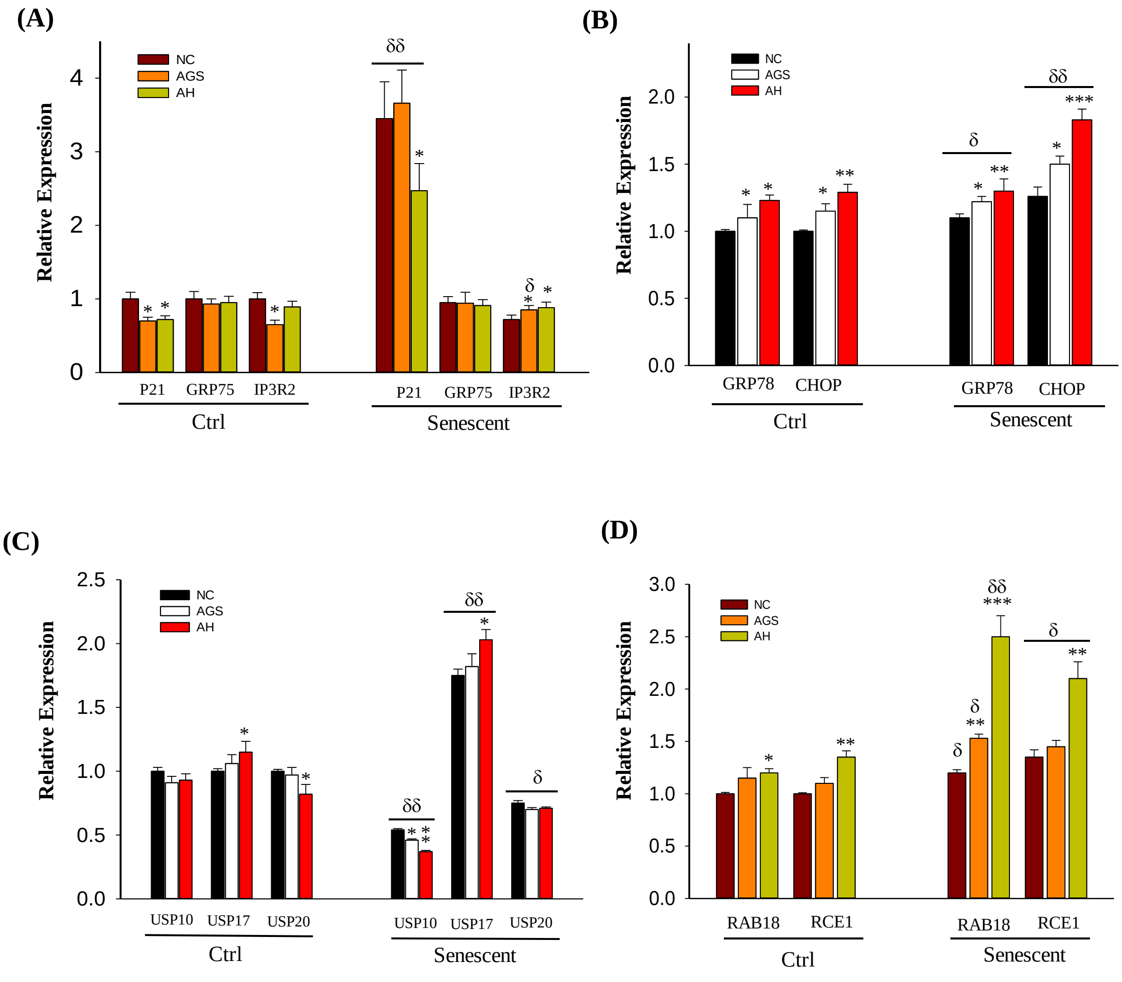

Figure S2 Cellular Stress Response in Non-senescent and Senescent HepG2 in Response to Ethanol and Cocaine Derivative Treatments. Quantitation of protein expression normalized with corresponding loading controls of tubulin and relative protein expression of non-senescent cells treated with vehicle was set as one; NC, control without drug or ethanol; AGS; cell incubated in an acetaldehyde generating system/medium consisting of ethanol, alcohol dehydrogenase (ADH) and nicotinamide adenine dinucleotide (NAD+); HA, hippuric acid (a cocaine derivative in the liver); AH, AGS plus hippuric acid; Western blots of selected metabolic proteins include: (**A**) P21, cyclin-dependent kinase inhibitor 1; GRP75, a member of the heat shock protein 70 gene family; IP3R2, the inositol 1,4,5-trisphosphate (IP3) receptor (IP3R) type 2; (**B**) GRP78, glucose-regulated protein 78; CHOP, DNA damage-inducible transcript 3, also known as C/EBP homologous protein; (**C**) USP10, deubiquitinase 10 involved in diverse cellular processes; USP17, ubiquitin-specific protease 17; USP20, ubiquitin specific peptidase 20; (**D**) RAB18, a member of the Rab family of Ras-related small GTPases; RCE1, Ras converting enzyme 1. *, $p<0.05$; **, $p<0.01$; ***, $p<0.005$ compared to NC; δ, $p<0.05$ and δδ, $p<0.01$ compared to control non-senescent cells.

Supplemental Figure S3

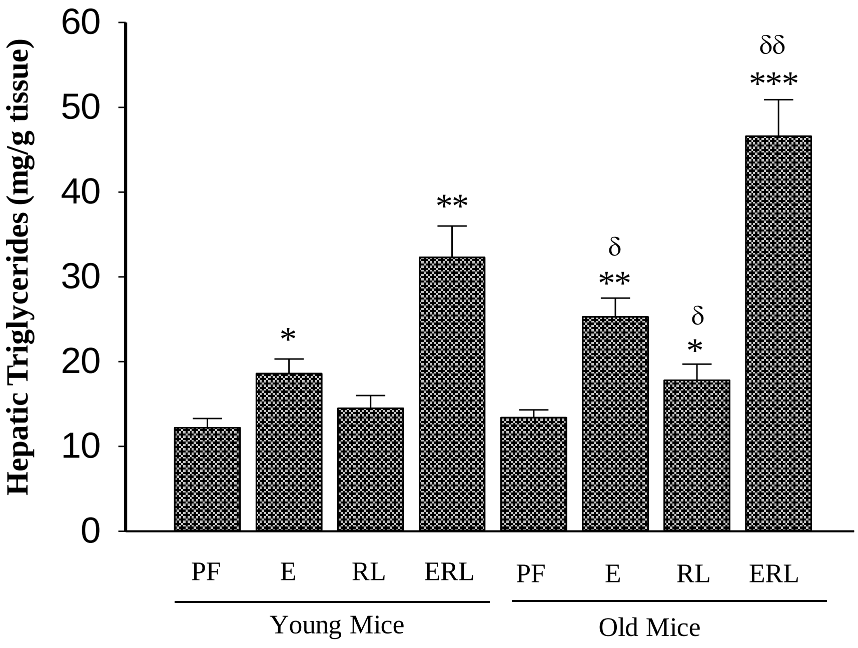

Figure S3 Hepatic Triglycerides in Young and Old Mice Treated with Ethanol and/or Anti-HIV Protease Inhibitors. PF, pair-fed control; E, fed with ethanol; RL, treated with ritonavir and lopinavir; ERL, fed ethanol and treated with ritonavir and lopinavir; *, $p<0.05$; **, $p<0.01$; ***, $p<0.005$ compared to PF in the same animal group; $^{\delta}$, $p<0.05$; ; $^{\delta\delta}$, $p<0.01$ compared between young and old mice.
